# Knowledge, Perceptions, and Attitudes of Israeli Healthcare Professionals Toward Mpox: A Survey-Based, Cross-Sectional Study

**DOI:** 10.3390/healthcare13070790

**Published:** 2025-04-01

**Authors:** Rola Khamisy-Farah, Raymond Farah, Hisham Atwan, Rabie Shehadeh, Inshirah Sgayer Shannan, Corinne Topchi, Yara Moallem, Basem Hijazi, Najim Z. Alshahrani, Woldegebriel Assefa Woldegerima, Nicola Luigi Bragazzi

**Affiliations:** 1 Clalit Health Services, Akko 13100, Israel; rkhamisy@yahoo.com; 2 Azrieli Faculty of Medicine, Bar-Ilan University, Safed 13100, Israel; raymond.f@ziv.health.gov.il (R.F.); rshehadeh1@gmail.com (R.S.); inshirah.sg.sh@gmail.com (I.S.S.); cori876@gmail.com (C.T.); mouallem.yara@gmail.com (Y.M.); basem.hijazi@biu.ac.il (B.H.); 3 Department of Internal Medicine B, Ziv Medical Center, Safed 13100, Israel; 4 Department of Internal Medicine, Kaplan Medical Centre, Hebrew University, Rehovot 76100, Israel; atwan.hisham@gmail.com; 5 Department of Obstetrics and Gynecology, Galilee Medical Center, Nahariya 22100, Israel; 6 Department of Family and Community Medicine, Faculty of Medicine, University of Jeddah, Jeddah 21589, Saudi Arabia; nalshahrani@uj.edu.sa; 7 Laboratory for Industrial and Applied Mathematics (LIAM), Department of Mathematics and Statistics, York University, Toronto, ON M3J 1P3, Canada; wassefaw@yorku.ca

**Keywords:** Mpox, healthcare professionals, knowledge, perceptions, emerging infectious diseases, public health, preparedness

## Abstract

**Background:** Mpox, a zoonotic viral disease, has recently emerged as a significant global public health challenge. Historically confined to endemic regions in West and Central Africa, recent outbreaks in non-endemic areas have highlighted the critical role of healthcare professionals (HCPs) in disease management and prevention. **Methods:** This cross-sectional study surveyed 709 Israeli HCPs, with a mean age of 40.6 ± 9.4 years, to evaluate their knowledge, perceptions, and attitudes toward mpox and identify gaps to inform educational and public health strategies. **Results:** Findings revealed that only 38.9% of respondents demonstrated good knowledge about mpox, while 61.1% exhibited poor knowledge. Misconceptions were prevalent: 37.9% identified paracetamol as a management option, with only 31.0% recognizing the need for antivirals. Notably, 67.1% correctly identified mpox as a viral disease, yet only 47.2% were aware that lymphadenopathy is a distinguishing symptom. Multivariable analysis identified several predictors of good knowledge, including marital status, being a medical doctor (versus an allied health professional), work seniority, and previous exposure to mpox-related information. Attitudes varied, with 57.7% expressing confidence in the ability of global health authorities to control mpox, and 59.0% expressing confidence in the Israeli Ministry of Health. Interest in learning more about mpox and related topics was high, with 67.4% showing interest in the epidemiology of emerging diseases. Respondents with good knowledge consistently exhibited more positive attitudes and confidence in managing mpox-related challenges. **Conclusions:** These findings underscore the urgent need for targeted educational interventions to enhance HCPs’ readiness and response capabilities. Strengthening professional training, incorporating emerging infectious diseases into curricula, and leveraging accurate media communication are critical steps toward improving preparedness for mpox and future outbreaks.

## 1. Introduction

Mpox, formerly known as monkeypox [1,2,3,4,5], is a zoonotic viral disease that has gained global prominence due to its potential for widespread transmission and the significant public health challenges it poses [6]. Historically confined to endemic regions in West and Central Africa [6], mpox has recently transcended geographical boundaries, with outbreaks reported in non-endemic areas. This shift underscores the urgent need for heightened global awareness, robust surveillance, and enhanced preparedness measures [7].

The most notable mpox outbreak occurred in 2022 when mpox spread globally beyond its traditionally affected regions [8]. Unlike previous outbreaks, which were primarily zoonotic or limited to localized human-to-human transmission, the 2022–2023 outbreak was driven largely by sexual networks, with rapid transmission reported in multiple non-endemic countries. This prompted the World Health Organization (WHO) to declare a Public Health Emergency of International Concern (PHEIC) [9], highlighting the urgency of a coordinated global response. The PHEIC was lifted in 2023 [10,11] following a sustained decline in infections. The response included targeted vaccination campaigns, behavioral interventions, and strengthened surveillance systems, which ultimately helped curb the spread of the disease.

However, a new outbreak in 2024 has reignited global concerns, particularly due to the emergence of a novel clade (Clade Ib) in the Democratic Republic of the Congo (DRC) [12]. This strain, which is exhibiting increased human-to-human transmission—especially through sexual contact—has led to an alarming surge in cases. Moreover, the virus has spread beyond the DRC into neighboring countries, including Burundi, Central African Republic, Kenya, the Republic of the Congo, Rwanda, Uganda, and Zambia [13], prompting the WHO to declare another PHEIC [14]. A few cases of Clade Ib mpox have been reported in non-African countries, including the United Kingdom, Ireland, Sweden, Germany, France, and Belgium [15], as well as the United States, Canada, China, India, Oman, Pakistan, and Thailand.

Unlike the 2022–2023 outbreak, which was predominantly seen in non-endemic regions, the 2024 resurgence is mostly centered in Africa, underscoring the need for sustained global support to combat neglected tropical diseases. The 2024 outbreak serves as a stark reminder that, without proactive measures and equitable access to medical countermeasures, such as vaccination, mpox remains a persistent global health threat [16].

Healthcare professionals play a pivotal role in combating infectious disease outbreaks, acting as the first line of defense through early detection, clinical management, and prevention efforts [17,18]. However, gaps in knowledge, widespread misconceptions, and varied attitudes among healthcare providers can compromise the effectiveness of responses to diseases like mpox [19,20,21,22,23]. A recent systematic review and meta-analysis revealed alarmingly low levels of awareness and preparedness among healthcare workers worldwide. The pooled estimate for good knowledge among healthcare workers was just 26.0% [95%CI 17.8–34.2], while the proportion with positive attitudes was only slightly higher (34.6% [95%CI 19.0–50.2]) [24].

Despite Israel being the most highly affected country in Asia and the first in the region to report an mpox case during the 2022–2023 mpox outbreak, little is known about the knowledge, perceptions, and attitudes of Israeli healthcare professionals regarding the disease. The outbreak in Israel began with the Health Ministry’s announcement of a suspected case on 20 May 2022, later confirmed on 21 May. By 21 June 2022, the country reported its first locally transmitted case, and to date 336 mpox cases have been documented [25,26].

This study aims to address this critical knowledge gap by examining the knowledge, attitudes, and perceptions of Israeli healthcare professionals toward mpox in the context of the 2022–2023 outbreak. By identifying educational needs and gaps, the study seeks to inform public health strategies and enhance training programs. Strengthening these areas is essential for bolstering the readiness and response capabilities of healthcare systems in managing mpox and other emerging infectious diseases.

## 2. Methods

### 2.1. Ethical Clearance

The study adhered to the principles of the Declaration of Helsinki. As the participants were healthcare workers, the local ethical committee of Bar-Ilan University, Safed, Israel, waived the requirement for formal ethical approval; however, informed written consent was obtained from all participants.

### 2.2. Study Design

A cross-sectional study was conducted from 1 March 2023 to 15 October 2024, to assess the knowledge, perceptions, and attitudes of Israeli healthcare workers toward mpox. As previously mentioned, the survey considered the 2022–2023 outbreak and took place before the resurgence of mpox and the emergence of the novel Clade Ib in non-African countries.

The study utilized a convenience sample, surveying participants across various demographic characteristics, professional backgrounds, and workplace settings. Data were collected using a structured questionnaire distributed via Google Forms, with outreach efforts supported by social media platforms and local healthcare organizations. The questionnaire captured variables such as age, gender, marital status, medical profession, workplace setting, duration of employment, and area of residence. Additionally, it included a series of questions on mpox knowledge, perceptions, and attitudes. These items were adapted from a validated questionnaire previously administered to 398 physicians in Saudi Arabia during a period of rising global mpox cases during the 2022–2023 outbreak [27]. Over 70% expressed interest in obtaining more knowledge about mpox, emerging diseases, and travel medicine. Female physicians, younger physicians (under 35), those working in the private sector, and those exposed to mpox information during medical training were significantly more likely to demonstrate good knowledge. Older physicians and those with no prior mpox education showed lower scores.

### 2.3. Study Questionnaire

The questionnaire consisted of two major sections. The former comprehensively evaluated understanding and perceptions of mpox across several dimensions, including prevalence, modes of transmission, clinical presentation, and management strategies. The first section addressed the geographic distribution of the disease, with questions focusing on its prevalence in the Middle East, Western and Central Africa, and Israel. These inquiries were designed to measure respondents’ awareness of the regional and global burden of mpox. The subsequent section examined the etiology and transmission pathways of the disease, distinguishing between viral and bacterial origins. It explored the zoonotic nature of mpox, particularly its potential for transmission through contact with infected animals, as well as its capacity for human-to-human spread. Questions also investigated the possibility of sexual transmission [28,29,30], with a focus on men who have sex with men (MSM), which is a group disproportionately affected by the current outbreak, as reflected by recent epidemiological data [31,32,33,34]. The other questions addressed overlapping clinical manifestations and critical distinguishing features between mpox and other infectious diseases, such as smallpox and chickenpox, from a comparative standpoint. One question focused on the presence of lymphadenopathy as a key diagnostic criterion to differentiate mpox from smallpox. The clinical signs and symptoms section assessed knowledge of the early manifestations of mpox, including flu-like symptoms, skin rashes, papules, vesicles, pustules, and gastrointestinal issues, such as diarrhea. This subsection aimed to evaluate familiarity with the disease’s progression and its defining diagnostic markers. The final part of the knowledge section of the questionnaire focused on management and prevention strategies. It explored the use of symptomatic treatments, such as paracetamol, the necessity of antivirals and antibiotics, and the availability of specific vaccines or treatments. Additional questions clarified misconceptions about immunity conferred by chickenpox vaccination and investigated the existence of dedicated interventions for mpox, aiming to enhance understanding of current medical approaches to its management.

The attitude section of the questionnaire aimed to assess respondents’ beliefs, feelings, and levels of interest regarding mpox and its broader implications. It included statements designed to gauge confidence in the ability of global and local health authorities to control the spread of mpox, as well as personal concerns about the potential for a worldwide epidemic. For instance, respondents were asked to evaluate their confidence in the global population’s capacity to manage the spread and the Israeli Ministry of Health’s effectiveness in taking localized action. The section also explored apprehensions about the potential health and societal impact of mpox, including whether respondents believed the virus could become a global epidemic, further escalating and imposing additional strain on already burdened healthcare systems, or posing a risk of spreading specifically within Israel. Additionally, it examined perceptions of the role of mass media in influencing prevention efforts and assessed interest in expanding knowledge about mpox, emerging diseases, and travel medicine. The final item evaluated perceptions of the risks associated with travel to regions experiencing mpox epidemics, reflecting concerns about the personal and public health implications of such travel.

### 2.4. Statistical Analysis

Descriptive statistics were performed to summarize the data. Data normality was checked through the Kolmogorov–Smirnov test. Continuous variables, such as age, were analyzed for mean, median, and interquartile range (IQR). Categorical variables, including gender, marital status, profession, workplace, and other survey items, were expressed as absolute and relative (percentages) numbers. The analysis focused on qualitatively and quantitatively appraising knowledge levels and attitudes toward mpox, including awareness of symptoms, transmission, treatment, vaccination, and risk perceptions, as previously mentioned. Responses to specific questions were analyzed to identify patterns and gaps in knowledge. An mpox knowledge score was constructed by summing correct replies, with the score dichotomized into “good knowledge” and “poor knowledge” categories. Respondents achieving 60% or more correct answers (15 out of 25) were classified as having good knowledge, while those below this threshold were categorized as having poor knowledge. This cut-off is commonly regarded as a passing grade, representing a foundational level of competency and ensuring that respondents demonstrated a substantial grasp of the key concepts being assessed while allowing for minor inaccuracies or gaps in knowledge. Higher thresholds may lead to an overly stringent classification excluding individuals with moderate knowledge, whereas a lower cutoff may be too lenient and fail to meaningfully distinguish between those with an adequate understanding and those lacking essential information. Furthermore, this cut-off is the same threshold that was employed in the validation study [27].

Additionally, an mpox attitude score was built by summing the replies to attitude-related questions and inverting the scores for negatively phrased items to ensure consistency in interpretation.

Univariate analyses were conducted to explore predictors of mpox knowledge and attitudes. Chi-square tests were employed to compare categorical variables, identifying significant associations between demographic characteristics and mpox knowledge levels. Mann–Whitney U or Kruskal–Wallis tests were used to examine relationships between demographic characteristics and mpox attitudes. Multivariable linear and logistic regression analyses were performed to identify predictors of good knowledge, with odds ratios (ORs) reported along with their 95% confidence intervals (CIs). Additionally, multivariable linear regression models were utilized to investigate the determinants of attitudes toward mpox and emerging global health challenges.

All calculations were performed using the open-source environment R (R Foundation for Statistical Computing, Vienna, Austria, 2023, accessible at https://www.R-project.org/) (accessed on 9 January 2025).

## 3. Results

### 3.1. Participants’ Characteristics

The study included 709 respondents aged 30 to 67 years, with a mean age of 40.6 ± 9.4 years and a median age of 38 years. Gender distribution was nearly equal, with 48.7% identifying as female and 51.3% as male. Regarding marital status, 56.7% of respondents were married, 37.5% were single, 5.4% were divorced, and 0.4% were widowed. In terms of geographic distribution of participants’ living places within Israel, the majority of respondents (60.9%) resided in the northern region, followed by 26.4% living in the central region. A smaller proportion, 12.9%, reported living in the southern region. Concerning the professional composition of the participants, 36.8% identified as allied health professionals and 63.2% as medical doctors. Workplace distribution indicated that 69.5% worked in hospitals, 27.4% in community settings, and 3.1% in both settings. Concerning work seniority, the largest proportion of respondents (38.8%) reported having more than five years of work experience, followed by 32.9% with one to five years of experience. Those with less than one year of work experience constituted 28.3% of the sample.

Further details are reported in Table 1, to which the reader is referred.

### 3.2. Exposure to Information About Mpox and Mpox Knowledge in Israeli Healthcare Workers

Survey responses highlighted varying levels of exposure to information about mpox. Notably, only 28.1% of participants reported receiving formal education about mpox during their studies, indicating a gap in academic coverage of the topic. In contrast, a substantial majority (76.7%) stated that they had heard of mpox at least once, suggesting that informal or external sources played a significant role in raising awareness about the disease.

The survey results assessing participants’ knowledge about mpox reveal varied levels of understanding across different aspects of the disease (Table 2). A majority of respondents correctly identified that mpox is not prevalent in Middle Eastern countries, with 79.1% answering “No”, while 61.4% recognized its prevalence in Western and Central Africa. A proportion of 18.8% incorrectly stated that mpox was widespread in Israel, suggesting limited knowledge about its local occurrence.

Regarding the nature of the disease, 67.1% correctly identified mpox as a viral infection, whereas 88.4% correctly ruled out bacterial etiology. However, misconceptions about transmission and symptomatology were observed. For example, 38.5% believed mpox is easily transmitted human-to-human, and 41.7% acknowledged transmission through a monkey bite. When asked whether travelers from America and Europe are the primary source of imported mpox cases, 37.2% responded affirmatively, while the majority (62.8%) correctly disagreed. Regarding similarities in symptoms between mpox and smallpox, 46.0% agreed that the two diseases share similar signs and symptoms, while 54.0% disagreed. Similarly, when asked about the resemblance between mpox and chickenpox, 45.1% believed they have similar symptoms, while 54.9% disagreed. Furthermore, 56.8% identified flu-like syndrome as an early symptom, while 59.5% associated rashes with mpox. A majority of respondents (70.4%) correctly identified that diarrhea is not a common sign or symptom of human mpox, while 29.6% mistakenly believed it to be associated with the disease. However, knowledge about more specific symptoms, such as papules (48.5%), vesicles (50.9%), and pustules (42.2%), was less definitive. Similarly, 47.2% understood that lymphadenopathy is a distinguishing feature between mpox and smallpox cases.

In terms of management, misconceptions about treatment options were evident. A proportion of 37.9% recognized paracetamol as a management option, while 31.0% believed antivirals were necessary. Antibiotics were correctly ruled out by 84.2% of participants. Similarly, only 35.8% were aware of a specific vaccine for mpox, with 18.9% stating that specific treatments exist. Misunderstandings about immunity and risk factors were also observed. A majority (67.1%) correctly stated that chickenpox vaccination does not confer immunity against mpox. However, fewer participants identified men who have sex with men as the primary risk group (34.6%), and only 36.8% acknowledged transmission through sexual intercourse.

Overall, mpox knowledge score was 14.7 ± 3.1 (median 15, IQR 5), with 38.9% and 61.1% of participants reporting good and poor knowledge levels, respectively.

### 3.3. Attitudes Toward Mpox and Emerging Global Health Challenges in Israeli Healthcare Workers

The results of the survey assessing attitudes toward mpox and related global health issues revealed diverse perspectives among respondents (Table 3). A majority expressed confidence in the global population’s ability to control the spread of mpox, with 42.2% agreeing and 15.5% strongly agreeing with this sentiment. Similarly, confidence in the Israeli Ministry of Health and the local population’s ability to manage the spread of mpox was comparably high, with 43.2% agreeing and 15.8% strongly agreeing. Concerns regarding mpox as a potential worldwide epidemic were evident in 23.7% of surveyees, with 13.0% strongly disagreeing and 29.8% disagreeing that it could escalate to a global scale. However, 33.6% remained neutral, indicating uncertainty or ambivalence. The strain on health systems posed by mpox was recognized by a substantial proportion of respondents, with 35.4% agreeing and 14.7% strongly agreeing, reflecting moderate apprehension about its potential impact on healthcare infrastructure in countries already impacted. The perception of mpox’s spread within Israel elicited mixed responses, with 31.6% agreeing and 8.9% strongly agreeing that it could occur, while 19.9% disagreed. Notably, media coverage was identified as a significant factor influencing global prevention efforts, with 42.0% agreeing and 16.5% strongly agreeing that mass media could play a critical role. Interest in learning more about mpox and related topics, including the epidemiology of emerging diseases and travel medicine, was high among respondents. A substantial proportion expressed a desire to gain further knowledge, with 39.6% agreeing and 25.7% strongly agreeing to learn more about mpox specifically. Similarly, interest in epidemiology and travel medicine was affirmed by 39.8% and 39.4% of respondents agreeing, respectively, and over a quarter strongly agreeing in both cases. Finally, a notable proportion of respondents perceived travel to countries with ongoing mpox epidemics as dangerous, with 36.4% agreeing and 15.8% strongly agreeing.

Overall, the mpox attitude score was 29.7 ± 5.7 (median 31, IQR 8.0), ranging from 10 to 45.

### 3.4. Determinants of Mpox Knowledge in Israeli Healthcare Workers

In the univariate analysis of the determinants of mpox knowledge (Table 4), age was not significantly associated with the level of knowledge. Among individuals with good knowledge, 44.6% were aged ≤38 years and 55.4% were aged >38 years, compared to 42.7% and 57.3% in the poor knowledge group, respectively (χ^2^ = 0.2, *p* = 0.630). Gender also did not show a significant association with knowledge levels. Males comprised 52.2% of the good knowledge group and 50.8% of the poor knowledge group, while females accounted for 47.8% and 49.2%, respectively (χ^2^ = 0.1, *p* = 0.723). Marital status demonstrated a statistically significant association with knowledge. Married individuals were more likely to have good knowledge (64.5%) compared to non-married individuals (35.5%). Conversely, in the poor knowledge group, married individuals constituted 51.7%, and non-married individuals accounted for 48.3% (χ^2^ = 11.2, *p* < 0.001). Profession significantly influenced knowledge levels. Among those with good knowledge, 83.3% were medical doctors, whereas 16.7% were allied health professionals. In contrast, only 50.3% of the poor knowledge group were medical doctors, while 49.7% were allied health professionals (χ^2^ = 77.4, *p* < 0.001). Workplace setting was significantly associated with knowledge levels. Hospital workers constituted 74.6% of the good knowledge group, compared to 66.3% of the poor knowledge group. Community-based professionals accounted for 21.0% of the good knowledge group and 31.4% of the poor knowledge group. Those working in both settings made up 4.3% and 2.3% of the respective groups (χ^2^ = 10.6, *p* = 0.005). Work seniority revealed a significant association. Individuals with less than one year of experience comprised 19.9% of the good knowledge group, compared to 33.7% in the poor knowledge group. Those with 1–5 years of experience made up 33.3% of the good knowledge group and 32.6% of the poor knowledge group. Professionals with more than five years of experience accounted for 46.7% of the good knowledge group and 33.7% of the poor knowledge group (χ^2^ = 18.7, *p* < 0.001). Area of residence was significantly associated with knowledge. In the good knowledge group, 69.6% resided in the North, 23.6% in the Center, and 6.9% in the South, whereas the poor knowledge group comprised 55.4% from the North, 27.9% from the Center, and 16.6% from the South (χ^2^ = 19.2, *p* < 0.001). Receiving information about mpox during studies was not significantly associated with knowledge. Among those with good knowledge, 28.6% reported having received information, compared to 27.7% in the poor knowledge group (χ^2^ = 0.0, *p* = 0.859). Hearing about mpox was, instead, significantly associated with knowledge. Among individuals with good knowledge, 87.0% had heard of mpox compared to 70.2% in the poor knowledge group (χ^2^ = 25.6, *p* < 0.001).

In the multivariable analysis of predictors of mpox knowledge (Table 5 and Table 6), age and gender did not significantly influence knowledge levels. Age showed a negligible effect (estimate = 0.002, SE = 0.013, *p* = 0.849; OR = 1.003, 95% CI [0.982, 1.025]). Similarly, no significant differences were observed between male and female healthcare professionals (estimate = −0.239, SE = 0.210, *p* = 0.256; OR = 0.787, 95% CI [0.537, 1.152]). In contrast, marital status emerged as a significant predictor. Non-married professionals exhibited lower knowledge levels compared to their married counterparts (estimate = −0.439, SE = 0.227, *p* = 0.054; OR = 5.727, 95% CI [3.645, 9.000]). Profession was a crucial determinant of mpox knowledge, with medical doctors demonstrating significantly greater knowledge compared to health-allied professionals (estimate = 2.323, SE = 0.255, *p* < 0.001; OR = 1.287, 95% CI [0.493, 3.362]). Workplace type influenced knowledge levels. While community-based professionals tended to exhibit lower knowledge (estimate = −0.026, SE = 0.640, *p* = 0.967; OR = 1.751, 95% CI [0.698, 4.393]), hospital-based professionals were more knowledgeable than those working in mixed settings (estimate = 0.415, SE = 0.617, *p* = 0.502; OR = 0.582, 95% CI [0.370, 0.917]). Work seniority was a significant predictor of knowledge. Professionals with less than one year of experience reported significantly lower knowledge compared to those with one to five years of experience (estimate = −0.684, SE = 0.269, *p* = 0.011; OR = 1.486, 95% CI [0.943, 2.342]). Conversely, those with more than five years of experience exhibited higher knowledge compared to the one-to-five-years group (estimate = 0.442, SE = 0.276, *p* = 0.109; OR = 1.068, 95% CI [0.708, 1.612]). Geographical location also influenced knowledge levels. Professionals in the South displayed lower knowledge compared to those in the Center (estimate = −0.499, SE = 0.359, *p* = 0.165; OR = 1.174, 95% CI [0.790, 1.747]). However, no significant differences were observed between professionals in the North and Center (estimate = −0.085, SE = 0.249, *p* = 0.734; OR = 0.702, 95% CI [0.364, 1.356]). Informational exposure during studies was not associated with higher knowledge levels (estimate = 0.021, SE = 0.241, *p* = 0.929; OR = 3.697, 95% CI [2.357, 5.799]). Lastly, prior awareness of mpox was a strong predictor of knowledge. Healthcare professionals who had previously heard of mpox demonstrated substantially higher knowledge levels (estimate = 1.685, SE = 0.256, *p* < 0.001; OR = 0.776, 95% CI [0.547, 1.100]).

### 3.5. Determinants of Attitudes Toward Mpox and Emerging Global Health Challenges in Israeli Healthcare Workers

In the univariate analysis, no significant difference was observed in mpox attitude scores between genders, with both male and female participants scoring an average of 29.7 ± 5.7 (*p* = 0.850). However, marital status significantly influenced scores (*p* = 0.002), with married participants reporting higher scores (30.3 ± 5.6) compared to their non-married counterparts (28.9 ± 5.7). Age was a borderline predictor (*p* = 0.117), as younger participants exhibited slightly higher scores (30.0 ± 5.8) than older ones (29.3 ± 5.6). Medical doctors had significantly higher scores (31.5 ± 5.1) compared to allied health professionals (26.6 ± 5.4), with a *p*-value <0.001. Work setting also played a role, with the highest scores observed among professionals working both in hospitals and in the community (34.7 ± 4.9), followed by those working exclusively in hospitals (29.9 ± 5.6) and those working solely in the community (28.7 ± 5.8), with a *p*-value <0.001. Work seniority was another significant factor (*p* = 0.008), as more senior professionals reported higher scores (30.6 ± 5.0) than those with less than one year of experience (28.7 ± 6.2) or with 1–5 years of experience (29.5 ± 5.9). Geographic disparities in mpox attitude scores were also identified (*p* < 0.001), with participants from the South scoring the lowest (26.5 ± 5.3), compared to those from the Center (29.1 ± 5.8) and the North (30.6 ± 5.5). Finally, healthcare workers with good knowledge exhibited significantly higher confidence in global efforts to control mpox (3.8 ± 0.8) compared to those with poor knowledge (3.4 ± 1.2), with a *p*-value <0.001. Similarly, confidence in the Israeli Ministry of Health’s ability to manage the spread of mpox was greater among those with good knowledge (3.9 ± 0.8) compared to those with poor knowledge (3.3 ± 1.1), also with a *p*-value <0.001. Concerns about mpox potentially becoming a worldwide epidemic were similar between the groups, with those with poor knowledge scoring slightly higher (2.8 ± 1.2) compared to those with good knowledge (2.7 ± 1.0), though this difference was not statistically significant (*p* = 0.190). However, beliefs about the strain mpox could place on health systems were significantly higher among workers with good knowledge (3.6 ± 1.0) than those with poor knowledge (3.2 ± 1.2), with a *p*-value <0.001. Perceptions of the likelihood of mpox spreading within Israel showed little difference between the groups, with workers with good knowledge scoring 3.2 ± 1.0 compared to 3.1 ± 1.1 for those with poor knowledge (*p* = 0.427). In contrast, confidence in the role of mass media in influencing global prevention was significantly higher among workers with good knowledge (3.9 ± 0.9) compared to those with poor knowledge (3.3 ± 1.2), with a *p*-value <0.001. Interest in learning about mpox and related topics was also greater among workers with good knowledge. Their interest in learning more about mpox was 4.0 ± 0.9 compared to 3.5 ± 1.3 for those with poor knowledge (*p* < 0.001). Similarly, their interest in the epidemiology of emerging diseases (4.0 ± 0.9 vs. 3.6 ± 1.2, *p* < 0.001) and travel medicine (3.9 ± 0.9 vs. 3.5 ± 1.3, *p* < 0.001) was significantly higher. Finally, perceptions of the danger of traveling to countries affected by mpox were slightly higher among workers with good knowledge (3.5 ± 1.1) compared to those with poor knowledge (3.2 ± 1.3), with a *p*-value of 0.011.

In the multivariable analysis of predictors of attitudes toward mpox and emerging global health challenges (Table 7), demographic predictors, including age, gender, and marital status, were not significant. Age had an estimate of −0.024 (SE = 0.023, *p* = 0.288), while the difference between males and females was −0.422 (SE = 0.381, *p* = 0.268). The comparison between non-married and married individuals showed an estimate of −0.199 (SE = 0.413, *p* = 0.630). Profession emerged as a significant predictor. Being a medical doctor compared to a health-allied professional was associated with an increase in attitudes score (estimate = 3.463, SE = 0.489, *p* < 0.001). Workplace setting also significantly influenced attitudes. Compared to those working in both community and hospital settings, individuals working only in community settings had a lower attitudes score (estimate = −2.332, SE = 1.159, *p* = 0.045), as did those working only in hospitals (estimate = −3.006, SE = 1.118, *p* = 0.007). Work seniority did not significantly predict attitudes. Comparisons between <1 year and 1–5 years (estimate = −0.324, SE = 0.490, *p* = 0.508) and >5 years and 1–5 years (estimate = 0.698, SE = 0.500, *p* = 0.164) showed no significant differences. Area of residence had minimal impact, with the South compared to the Center showing a borderline non-significant trend toward lower attitude scores (estimate = −1.269, SE = 0.651, *p* = 0.051), while no differences between the North and the Center could be found (estimate = 0.398, SE = 0.451, *p* = 0.378). Receiving information about mpox during studies significantly predicted lower attitude scores, with an estimate of −1.048 (SE = 0.437, *p* = 0.017). Prior awareness of mpox did not significantly affect attitudes (estimate = 0.297, SE = 0.478, *p* = 0.535). Finally, higher knowledge scores about mpox were significantly associated with more positive attitudes (estimate = 0.418, SE = 0.069, *p* < 0.001).

## 4. Discussion

The findings of this study revealed significant gaps in knowledge and perceptions of mpox among Israeli healthcare professionals, despite their critical role in managing infectious disease outbreaks. The results highlighted the need for targeted educational interventions to enhance awareness and preparedness.

Less than half of the participants reported receiving information about mpox during their studies, pointing to a need for improved educational curricula that address emerging diseases. Misconceptions, such as the belief that antibiotics are necessary for treating mpox, further emphasize the importance of targeted professional training. Interestingly, while a majority of respondents demonstrated awareness of some clinical features of mpox, fewer recognized the unique signs that differentiate it from other diseases, such as swollen lymph nodes. This highlights the need for enhanced training to improve diagnostic capabilities. On the other hand, the study also revealed a strong interest among participants in learning more about mpox and the epidemiology of emerging diseases, suggesting an opportunity to leverage this enthusiasm for professional development programs. Furthermore, perceptions of risk varied, with many participants acknowledging the potential strain on health systems and the influence of media in shaping public attitudes.

Of note, healthcare workers with good knowledge demonstrated significantly greater confidence, awareness, and interest in mpox-related topics compared to their counterparts with poor knowledge. These findings highlight the importance of targeted education and training to enhance knowledge among healthcare workers, fostering a more informed and proactive response to global health challenges like mpox.

Our study found that Israeli healthcare professionals exhibited higher levels of knowledge and more positive attitudes toward mpox compared to global averages [24]. Specifically, 38.9% of Israeli healthcare workers were classified as having good knowledge, surpassing the global meta-analysis estimate of 26.0%. Their knowledge levels were also higher than those reported in Africa (17.1%), Asia (26.1%), and Europe (32.5%), though still lower than in the United States (46.7%). Similarly, positive attitudes toward Mpox control were observed in 57.7% and 59.0% of Israeli respondents regarding global and local health authorities, respectively, compared to the global average of 34.6%.

In the questionnaire validation study [27], 55% of physicians scored well (≥14/23). Most physicians (94.7%) recognized mpox as a viral disease, but gaps were evident, with only 50.6% knowing that antiviral treatments are required. A proportion of 15.3% incorrectly suggested antibiotics for treatment. Many struggled to identify differentiating symptoms, such as lymphadenopathy (correct in 21.1%); 78.6% believed Saudi Arabia could control mpox locally, but fewer (56%) felt the same globally; 64.6% acknowledged its potential transmissibility to Saudi Arabia; 59.3% deemed travel to endemic regions risky.

Furthermore, in our study, multivariable analysis identified several predictors of good knowledge, including marital status, being a medical doctor (versus an allied health professional), work seniority, and previous exposure to mpox-related information, aligning with the global meta-analysis that reported 41.6% good knowledge among healthcare workers with longer experience. Gender, however, was not a significant predictor in either study, with similar knowledge and attitude levels reported across male and female respondents. Regional differences within Israel, such as higher knowledge among northern healthcare professionals, further emphasize the influence of geographical and professional contexts. Despite these relatively strong results, gaps remain—only 28.1% of Israeli respondents reported receiving formal education regarding mpox, underscoring the need for targeted training. The findings highlight the need for targeted educational interventions to improve healthcare professionals’ preparedness and response.

As such, our findings have important implications for public health strategies. Educational interventions should focus on addressing specific knowledge gaps and correcting misconceptions [35]. Additionally, policymakers should prioritize the inclusion of emerging infectious diseases in professional training programs and promote accurate, evidence-based media communication to enhance public awareness and trust. Overall, the present study underscores the urgent need for a coordinated effort to enhance healthcare professionals’ preparedness for mpox and other emerging infectious diseases. Future research should explore the impact of targeted educational interventions and examine how improved knowledge translates into clinical practice and public health outcomes.

## 5. Strengths and Limitations

This study has several key strengths, foremost among them its novel focus on an underexplored yet highly relevant public health issue. To the best of our knowledge, this is the first study conducted in Israel examining the knowledge and attitudes of healthcare workers toward mpox. By addressing this critical gap, the research provides valuable insights specific to the Israeli healthcare system while contributing to the broader global discourse on mpox preparedness. Additionally, the study employs a validated and structured questionnaire, ensuring methodological rigor, and includes a diverse sample of healthcare professionals, enhancing the representativeness of perspectives. The large sample size and comprehensive analysis further strengthen the robustness of the findings.

However, certain limitations should be acknowledged. The use of a convenience sample rather than a randomized sampling approach may affect the generalizability of the results and introduce selection bias. Moreover, the cross-sectional design limits causal inferences, and the reliance on self-reported data raises the potential for biases, such as social desirability bias. While multiple regression models were applied, some key variables—such as prior exposure to mpox patients and participation in institutional training programs—were not explored in depth, potentially introducing confounding effects.

Despite these limitations, this study represents a foundational effort in understanding Israeli healthcare workers’ perspectives on mpox and offers critical insights for future research and public health initiatives.

## 6. Future Directions

Future research should address the limitations of this study by employing longitudinal designs to examine changes in knowledge and attitudes over time. Expanding the study to include other countries and healthcare settings could enhance generalizability. Furthermore, interventional studies are needed to evaluate the effectiveness of targeted educational programs on improving knowledge and preparedness. Finally, investigating the role of digital tools and media campaigns in disseminating accurate information about mpox could also be valuable.

## 7. Conclusions

This study highlights significant gaps in knowledge and varying perceptions of mpox among healthcare professionals, with practical implications for policymakers and medical educators. Our findings underscore the importance of targeted educational interventions to enhance preparedness and response capabilities. Strengthening professional training programs by integrating mpox education into medical curricula is essential for mitigating the spread of mpox as well as other emerging infectious diseases. By addressing these gaps, healthcare systems can be better equipped to manage future outbreaks and protect public health effectively.

## Figures and Tables

**Table 1:** Demographic and professional characteristics of survey respondents.

Variable	Value
Age (mean ± SD; median [IQR])	40.6 ± 9.4; 38 [11.0]
Gender (*n*, %)	
Male	364 (51.3%)
Female	345 (48.7%)
Marital status (*n*, %)	
Single	266 (37.5%)
Married	402 (56.7%)
Divorced	38 (5.4%)
Widowed	3 (0.4%)
Living place (*n*, %)	
North	432 (60.9%)
Center	186 (26.4%)
South	91 (12.9%)
Profession (*n*, %)	
Medical doctor	448 (63.2%)
Allied health professional	261 (36.8%)
Work place (*n*, %)	
Hospital	493 (69.5%)
Community	194 (27.4%)
Both	22 (3.1%)
Work seniority (*n*, %)	
<1 year	201 (28.3%)
1–5 years	233 (32.9%)
>5 years	275 (38.8%)

**Table 2:** Distribution of responses to knowledge items related to mpox.

Knowledge Item	Yes [*n* (%)]	No [*n* (%)]
Q1. Is mpox prevalent in Middle Eastern countries?	148 (20.9)	561 (79.1)
Q2. Is mpox prevalent in Western and Central Africa?	435 (61.4)	274 (38.6)
Q3. Are there many human mpox cases in Israel?	133 (18.8)	576 (81.2)
Q4. Is mpox a viral disease infection?	476 (67.1)	233 (32.9)
Q5. Is mpox a bacterial disease infection?	82 (11.6)	627 (88.4)
Q6. Is mpox easily transmitted human-to-human?	273 (38.5)	436 (61.5)
Q7. Could mpox be transmitted through a bite of an infected monkey?	296 (41.7)	413 (58.3)
Q8. Are travelers from America and Europe the primary source of imported cases of mpox?	264 (37.2)	445 (62.8)
Q9. Do mpox and smallpox have similar signs and symptoms?	326 (46.0)	383 (54.0)
Q10. Do mpox and chickenpox have similar signs and symptoms?	320 (45.1)	389 (54.9)
Q11. Is a flu-like syndrome one of the early signs or symptoms of human mpox?	403 (56.8)	306 (43.2)
Q12. Are rashes on the skin one of the signs or symptoms of human mpox?	422 (59.5)	287 (40.5)
Q13. Are papules on the skin one of the signs or symptoms of human mpox?	344 (48.5)	365 (51.5)
Q14. Are vesicles on the skin one of the signs or symptoms of human mpox?	361 (50.9)	348 (49.1)
Q15. Are pustules on the skin one of the signs or symptoms of human mpox?	299 (42.2)	410 (57.8)
Q16. Is diarrhea one of the signs or symptoms of human mpox?	210 (29.6)	499 (70.4)
Q17. Is lymphadenopathy (swollen lymph nodes) one clinical sign or symptom that could be used to differentiate between mpox and smallpox cases?	335 (47.2)	374 (52.8)
Q18. One management option for symptomatic mpox patients is to use paracetamol	269 (37.9)	440 (62.1)
Q19. Are antivirals required in the management of human mpox patients?	220 (31.0)	489 (69.0)
Q20. Are antibiotics required in the management of human mpox patients?	112 (15.8)	597 (84.2)
Q21. Are people who had the chickenpox vaccine immunized against mpox?	233 (32.9)	476 (67.1)
Q22. Is there a specific vaccine for mpox?	254 (35.8)	455 (64.2)
Q23. Is there a specific treatment for mpox?	134 (18.9)	575 (81.1)
Q24: Can mpox be transmitted from person to person through sexual intercourse?	261 (36.8)	448 (63.2)
Q25: Are men who have sex with men the primary risk group for mpox?	245 (34.6)	464 (65.4)

**Table 3:** Distribution of responses to attitude items related to mpox and emerging diseases.

Attitude Item	Strongly Disagree [*n* (%)]	Disagree [*n* (%)]	Neutral [*n* (%)]	Agree [*n* (%)]	Strongly Agree [*n* (%)]
A1: I am confident that the world’s population can control the spread of mpox around the world	41 (5.8)	70 (9.9)	189 (26.7)	299 (42.2)	110 (15.5)
A2: I am sure that the Israeli Ministry of Health and the local population can take local control against the spread of mpox	44 (6.2)	61 (8.6)	186 (26.2)	306 (43.2)	112 (15.8)
A3: I have a bad feeling that the mpox virus could become a worldwide epidemic	92 (13.0)	211 (29.8)	238 (33.6)	122 (17.2)	46 (6.5)
A4: I think mpox could add a new strain on the health system in countries already affected	60 (8.5)	106 (15.0)	188 (26.5)	251 (35.4)	104 (14.7)
A5: I think mpox can spread in Israel	60 (8.5)	141 (19.9)	221 (31.2)	224 (31.6)	63 (8.9)
A6: I think mass media coverage of mpox could have an impact on global prevention	51 (7.2)	63 (8.9)	180 (25.4)	298 (42.0)	117 (16.5)
A7: I am interested in learning more about mpox	47 (6.6)	65 (9.2)	134 (18.9)	281 (39.6)	182 (25.7)
A8: I am interested in learning more about the epidemiology of emerging diseases	45 (6.3)	51 (7.2)	135 (19.0)	282 (39.8)	196 (27.6)
A9: I am interested in learning more about travel medicine	50 (7.1)	61 (8.6)	149 (21.0)	279 (39.4)	170 (24.0)
A10: I think it’s dangerous to go to countries with an epidemic of mpox	66 (9.3)	110 (15.5)	163 (23.0)	258 (36.4)	112 (15.8)

**Table 4:** Univariate analysis of the predictors of mpox knowledge in healthcare professionals.

Variable	Good Knowledge	Poor Knowledge	Statistical Significance
Age			χ^2^ = 0.2, *p* = 0.630
≤38 y	123 (44.6%)	185 (42.7%)	
>38 y	153 (55.4%)	248 (57.3%)	
Gender			χ^2^ = 0.1, *p* = 0.723
Male	144 (52.2%)	220 (50.8%)	
Female	132 (47.8%)	213 (49.2%)	
Marital status			χ^2^ = 11.2, *p* < 0.001
Married	178 (64.5%)	224 (51.7%)	
Non married	98 (35.5%)	209 (48.3%)	
Profession			χ^2^ = 77.4, *p* < 0.001
Medical doctor	230 (83.3%)	218 (50.3%)	
Allied health professional	46 (16.7%)	215 (49.7%)	
Workplace			χ^2^ = 10.6, *p* = 0.005
Hospital	206 (74.6%)	287 (66.3%)	
Community	58 (21.0%)	136 (31.4%)	
Both	12 (4.3%)	10 (2.3%)	
Work seniority			χ^2^ = 18.7, *p* < 0.001
<1 year	55 (19.9%)	146 (33.7%)	
1–5 years	92 (33.3%)	141 (32.6%)	
>5 years	129 (46.7%)	146 (33.7%)	
Area of Residence			χ^2^ = 19.2, *p* < 0.001
North	192 (69.6%)	240 (55.4%)	
Center	65 (23.6%)	121 (27.9%)	
South	19 (6.9%)	72 (16.6%)	
Information about mpox during studies Yes No	79 (28.6%) 197 (71.4%)	120 (27.7%) 313 (72.3%)	χ^2^ = 0.0, *p* = 0.859
Ever heard of mpox			χ^2^ = 25.6, *p* < 0.001
Yes	240 (87.0%)	304 (70.2%)	
No	36 (13.0%)	129 (29.8%)	

**Table 5:** Multivariable linear regression analysis shedding light on the predictors of mpox knowledge in healthcare professionals.

Predictor	Estimate	SE	T	*p*	Standardized Estimate	95%CI Lower Bound	95%CI Upper Bound
Intercept	11.997	0.900	13.334	< 0.001			
Age	0.002	0.013	0.191	0.849	0.007	−0.068	0.082
Gender							
Male—Female	−0.239	0.210	−1.136	0.256	−0.077	−0.211	0.056
Marital status							
Non married–Married	−0.439	0.227	−1.933	0.054	−0.142	−0.287	0.002
Profession							
Medical doctor–Health Allied professional	2.323	0.255	9.098	< 0.001	0.754	0.591	0.916
Work place							
Community–Both	−0.026	0.640	−0.041	0.967	−0.008	−0.416	0.399
Hospital–Both	0.415	0.617	0.672	0.502	0.135	−0.259	0.528
Work seniority							
<1 year–1–5 years	−0.684	0.269	−2.540	0.011	−0.222	−0.394	−0.050
>5 years–1–5 years	0.442	0.276	1.604	0.109	0.144	−0.032	0.319
Area of Residence							
North–Center	−0.085	0.249	−0.340	0.734	−0.027	−0.186	0.131
South–Center	−0.499	0.359	−1.391	0.165	−0.162	−0.391	0.067
Information about mpox during studies							
Yes–No	0.021	0.241	0.087	0.929	0.007	−0.147	0.161
Ever heard of mpox							
Yes–No	1.685	0.256	6.572	<0 .001	0.547	0.383	0.710

**Table 6:** Multivariable logistic regression analysis shedding light on the predictors of mpox knowledge in healthcare professionals.

Predictor	Estimate	SE	Z	*P*	Odds-Ratio	95%CI Lower Bound	95%CI Upper Bound
Intercept	−3.085	0.756	−4.079	<0.001	0.046	0.010	0.201
Age	0.003	0.011	0.270	0.787	1.003	0.982	1.025
Gender							
Male–Female	−0.240	0.195	−1.234	0.217	0.787	0.537	1.152
Marital status							
Non married–Married	1.745	0.231	7.568	<0.001	5.727	3.645	9.000
Profession							
Medical doctor–Health Allied professional	0.253	0.490	0.516	0.606	1.287	0.493	3.362
Work place							
Community–Both	0.560	0.469	1.194	0.232	1.751	0.698	4.393
Hospital–Both	−0.541	0.232	−2.334	0.020	0.582	0.370	0.917
Work seniority							
<1 year–1–5 years	0.396	0.232	1.707	0.088	1.486	0.943	2.342
>5 years–1–5 years	0.066	0.210	0.314	0.754	1.068	0.708	1.612
Area of Residence							
North–Center	−0.354	0.336	−1.054	0.292	0.702	0.364	1.356
South–Center	0.161	0.203	0.793	0.428	1.174	0.790	1.747
Information about mpox during studies							
Yes–No	1.308	0.230	5.693	<0.001	3.697	2.357	5.799
Ever heard of mpox							
Yes–No	−0.254	0.178	−1.423	0.155	0.776	0.547	1.100

**Table 7:** Multivariable linear regression analysis shedding light on the predictors of attitudes toward mpox in Israeli healthcare professionals.

Predictor	Estimate	SE	T	*P*	Standardized Estimate	95%CI Lower Bound	95%CI Upper Bound
Intercept	25.200	1.825	13.809	<0.001			
Age	−0.024	0.023	−1.063	0.288	−0.040	−0.113	0.034
Gender							
Male–Female	−0.422	0.381	−1.108	0.268	−0.074	−0.205	0.057
Marital status							
Non-married–Married	−0.199	0.413	−0.482	0.630	−0.035	−0.177	0.107
Profession							
Medical doctor–Health Allied professional	3.463	0.489	7.081	<0.001	0.606	0.438	0.774
Workplace							
Community–Both	−2.332	1.159	−2.013	0.045	−0.408	−0.807	−0.010
Hospital–Both	−3.006	1.118	−2.689	0.007	−0.526	−0.911	−0.142
Work seniority							
<1 year–1–5 years	−0.324	0.490	−0.662	0.508	−0.057	−0.225	0.112
>5 years–1–5 years	0.698	0.500	1.395	0.164	0.122	−0.050	0.294
Area of Residence							
North–Center	0.398	0.451	0.882	0.378	0.070	−0.085	0.224
South–Center	−1.269	0.651	−1.951	0.051	−0.222	−0.446	0.001
Information about mpox during studies							
Yes–No	−1.048	0.437	−2.397	0.017	−0.183	−0.334	−0.033
Ever heard of mpox							
Yes–No	0.297	0.478	0.621	0.535	0.052	−0.112	0.216
Mpox knowledge score	0.418	0.069	6.088	<0.001	0.225	0.153	0.298

## Data Availability

All generated data is reported in the present paper.
